# Contactless Manipulation of Soft Robots

**DOI:** 10.3390/ma12193065

**Published:** 2019-09-20

**Authors:** Jae Gwang Kim, Jeong Eun Park, Sukyoung Won, Jisoo Jeon, Jeong Jae Wie

**Affiliations:** Department of Polymer Science and Engineering, Inha University, Incheon 22212, Korea

**Keywords:** soft robots, anisotropic materials, liquid crystalline polymers, magnetic composites, hydrogels

## Abstract

In recent years, jointless soft robots have demonstrated various curvilinear motions unlike conventional robotic systems requiring complex mechanical joints and electrical design principles. The materials employed to construct soft robots are mainly programmable anisotropic polymeric materials to achieve contactless manipulation of miniaturized and lightweight soft robots through their anisotropic strain responsivity to external stimuli. Although reviews on soft actuators are extensive, those on untethered soft robots are scant. In this study, we focus on the recent progress in the manipulation of untethered soft robots upon receiving external stimuli such as magnetic fields, light, humidity, and organic solvents. For each external stimulus, we provide an overview of the working principles along with the characteristics of programmable anisotropic materials and polymeric composites used in soft robotic systems. In addition, potential applications for untethered soft robots are discussed based on the physicochemical properties of programmable anisotropic materials for the given external stimuli.

## 1. Introduction

**Figure 1:**
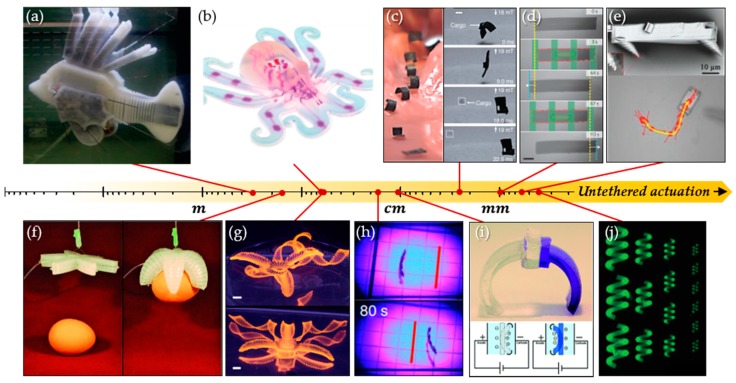
Soft robots (from meter to sub-mm). (**a**) Vascular-system-adapted swimming soft robot. (**b**) Chemical-reaction-fueled pneumatic octobot. (**c**) Multi-modal magnetic soft robot. (**d**) Underwater swimmer with a photoactive liquid crystal polymer. (**e**) Walking liquid crystal polymeric soft robot. (**f**) Pneumatic gripper. (**g**) Biomimetic three-dimensional (3D)-printed hydrogel flower. (**h**) Helical photomobile liquid crystal polymeric soft robot. (**i**) Electro-active walking soft robot. (**j**) Magnetic swimmer. (**a**) Reproduced with permission [55]. Copyright 2019, Nature Publishing Group. (**b**) Reproduced with permission [43]. Copyright 2016, Nature Publishing Group. (**c**) Reproduced with permission [9]. Copyright 2018, Nature Publishing Group. (**d**) Reproduced with permission [26]. Copyright 2016, Nature Publishing Group. (**e**) Reproduced with permission [19]. Copyright 2015, Wiley-VCH. (**f**) Reproduced with permission [56]. Copyright 2011, Wiley-VCH. (**g**) Reproduced with permission [30]. Copyright 2016, Nature Publishing Group. (**i**) Reproduced with permission [40]. Copyright 2014, Royal Society of Chemistry. (**j**) Reproduced with permission [57]. Copyright 2018, Wiley-VCH.

Soft robots implement facile curvilinear motions and various functions including bending, twisting, and folding without employing numerous rigid joints. In particular, untethered soft robotic systems provide a high degree of freedom for motility by removing limitations of working distance and complexity of motion originating from distance and wire tangling, respectively. In the case of centimeter-scale or larger bulk soft robots, the pneumatic actuation mechanism is dominantly demonstrated through air pressure control. However, these pneumatic systems often utilize a tethered inlet and outlet of fluid, thus hampering miniaturization and limiting the degree of freedom for soft robotic motility. To achieve untethered manipulation of miniaturized soft robots, diverse anisotropic materials and composites have been investigated for stimuli-responsive motility using magnetic fields [1,2,3,4,5,6,7,8,9,10,11], light [12,13,14,15,16,17,18,19,20,21,22,23,24,25,26,27], organic solvents [28,29,30,31,32,33,34,35,36,37,38], and chemical reactions [39,40,41,42,43]. As representative anisotropic materials, liquid crystalline polymers can achieve contactless motility under external stimuli through programmable molecular orientations and resultant deformation. In particular, photoactive molecular machines that perform reversible molecular contraction and expansion by actinic light can be polymerized together with liquid crystalline monomers to produce photomotility [12,13,14,15,16,17,18,19,20,21,22,23,24,25,26,27]. Magnetic composites allow continuous and multimodal motility through programming particle alignment and/or the magnitude and direction of electromagnetic fields [1,2,3,4,5,6,7,8,9,10,11]. Moreover, hygromorphic materials and hydrogels can also show directed motility in response to chemical (solvent, water, and pH) stimuli [28,29,30,31,32,33,34,35,36,37,38]. Recent research reports examples of soft actuators constructed from the aforementioned stimuli-responsive materials including polyelectrolyte and shape memory polymers [44,45,46]. In addition, reviews on anisotropic soft materials have been restricted to actuators or addressed tethered and untethered soft robotic systems [47,48,49,50,51,52,53,54]. As shown in Figure 1, the merits of anisotropic materials can be prominent as soft robots move toward small-scale contactless manipulation, as previously mentioned. Therefore, the aim of this study is to highlight recent reports and introduce the working principles for each material whereby external stimuli are converted into mechanical energy (Table 1). More specifically, the system using anisotropic magnetic particles and polymer composites is discussed in terms of robot geometry and particle alignment, and the various movements of liquid crystalline polymers and composites, which are representative examples of anisotropic molecules, are also considered in this work. Finally, we discuss the responsivity of soft robots to solvents, pH, or chemical reactions, and we conclude by suggesting from an interdisciplinary perspective future research to overcome the remaining challenges.

## 2. Magnetic Soft Robots

Magnetic actuation has been significantly considered in battery-free systems and blocked environments because of the penetrable feature of a magnetic field. An external magnetic stimulus manipulates the locomotion of soft robots with a fast response and endows a powerful functional tool for actuation without setting the atmospheric pressure, temperature, solvent, or pH conditions. 

In addition, its non-destructive property is also advantageous to extend soft robotic applications to biomedical technologies for drug delivery, diagnosis, and non-surgical therapy.

Diverse modes of magnetomotility have been reported, including swimming [1,2,4], crawling [5,6], rolling [7], tumbling [8], and multimodal motilities [9,10]. Motile strategies to achieve magnetic maneuvers have been widely developed by programming (i) the geometry of robots, (ii) the positional alignments of magnetic components, and (iii) the polarity of the magnetic domain of magnetic components. With respect to magnetic materials, the driving force of motility is a magnetic torque to match the magnetic alignments or polarity in the direction of the magnetic field. Positional alignments can be induced by a relatively weak magnetic field of permanent magnets (a few hundreds of mT) or a solenoid coil such as a Helmholtz coil (a few tens of mT). However, programming polarity requires strong electromagnetic devices (a few T) that have an iron core wrapped in wire. In addition to preparing magnetic soft robots, the Helmholtz coil system has the advantage of simultaneous control of three axes to manipulate the magneto-motility of soft robots. In the case of programmed magnetic polarity, selective control can achieve multimodal motility. However, in a permanent setup, precisely changing the magnetic direction is more difficult. By contrast, the Helmholtz coil system restricts the moving distances of soft robots because of the constrained space of the device, unlike permanent magnets.

The magnetic characteristics of magnetic materials are also major factors for on-demand magnetic actuation. Magnetism is generally categorized into diamagnetism, paramagnetism, ferromagnetism, and ferrimagnetism based on the variation in dipole moments under an external magnetic field. Diamagnetic materials have oppositely aligned dipole moments to the direction of the magnetic field, although the dipole moments vanish in the absence of a magnetic field. Paramagnetic materials have aligned dipole moments in the direction of the magnetic field. Then, they are varied to randomized alignments without a magnetic field. Finally, ferromagnetic materials have aligned dipole moments in the direction of the magnetic field, which are retained even when the magnetic field is removed [10]. Ferrimagnetic materials have a similar property of ferromagnetism except that they contain diamagnetic elements such as oxygen, which results in reduced magnetization. 

**Figure 2:**
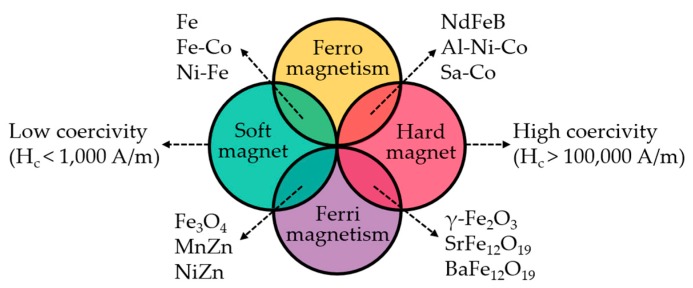
Classification of magnetic materials.

The ferro- and ferri-magnetic particles are divided into two types, soft and hard magnets, using a criterion of magnetic coercivity (Figure 2). According to the International Electrotechnical Commission Standard 404-1, the coercivity (H_c_) of soft magnets is less than 1000 A/m, whereas that of hard magnets is greater than 100,000 A/m [58]. The residual magnetization (M_r_) of hard magnets is relatively higher than that of soft magnets, leading to the tunability of dipole moments via electromagnetic devices. In addition, a superparamagnetic feature appears in ferro- and ferri-magnetic particles [59]. The coercivity and remanence are zero when the particle diameter decreases to 10–20 nm. Thermal fluctuation causes the magnetic moment to flip because the thermal energy is higher than the energy barrier for magnetization. Superparamagnetism appears in the randomized direction of the magnetization vector in the absence of an external magnetic field. Superparamagnetic particles do not retain magnetization in the absence of a magnetic field, decreasing the probability of aggregation. In the field of soft robotics, superparamagnetic magnetite is utilized to accomplish magnetic actuation without magneto-hysteresis.

Modifying magnetic particles can be a strategy to enhance magnetic response. A combination of magnetic particles generates a synergistic effect such as enhancements of storage modulus in aspects of magnetorheological properties [60,61,62,63,64]. Exchange coupling leads to reduced saturated magnetization (M_s_) and enhanced coercivity (H_c_). Composite particles dispersed in a polymer matrix can be an alternative to producing a higher magneto-response under a lower magnetic field in the field of magnetically active soft robots.

### 2.1. Programming Geometry of Robots for Desired Actuation

**Figure 3:**
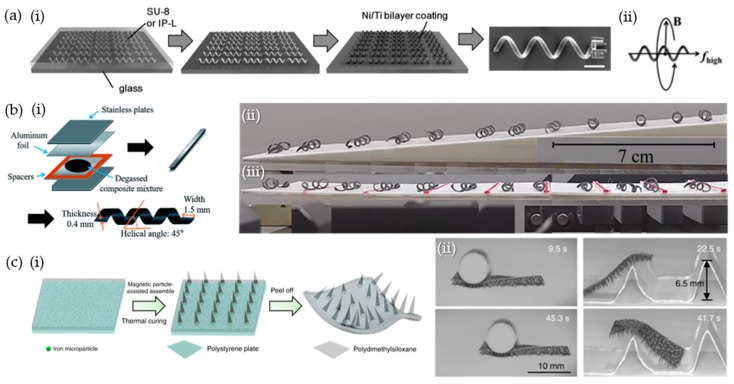
Geometry-induced locomotion of magnetic soft robots. (**a**) Helical soft robots produced using the dynamic light writing process when a photoresist polymer is used, followed by Ni/Ti bilayer coating: (**i**) scanning electron microscopy (SEM) image of helical micromachines with a microholder (scale bar: 10 μm), and (**ii**) principle of corkscrew motion. (**b**) Fabrication scheme for molding a helical geometry via (**i**) two-step polymerization, and (**ii**) ascending performance on uphill obstacles, and (**iii**) discrete walls. (**c**) (**i**) Preparation of multi-legged soft robots, (**ii**) loading performance at 100 times its own weight and when overcoming an obstacle. (**a**) was reproduced with permission [1]. Copyright 2012, Wiley-VCH.

Helical geometry has been mainly reported to achieve 3D swimming in liquid environments inspired by bacterial flagella [1,2,3,11]. The rotational motion of the helical robots was converted to linear translational motion such as a corkscrew motion, which is an effective strategy for propulsion at a low Reynolds number. The helical 3D structure can be prepared using a top-down approach such as dynamic light writing (DLW) [1]. A helical geometry was fabricated using a SU-8 or IP-L, followed by a coating of an Ni/Ti bilayer (Figure 3(ai)). SU-8 polymer is a commonly used epoxy-based negative photoresist material. The helix angle is crucial in achieving corkscrew motion and reducing wobbling from the rotational axis at the high frequency of a magnetic field (Figure 3(aii)). Transportation functionality of a micro holder was demonstrated by loading and releasing polystyrene particles with a diameter of 6 μm. A microparticle was loaded by the fluidic drag force against a swimming motion and released by reversed swimming backward. The capability of transportation implies the potential of biomedical applications including cancer therapy with localized drug delivery. 

Dip-coating of magnetite (Fe_3_O_4_) was conducted on the helical frame of *Spirulina platensis* bacteria to readily prepare 3D swimming soft robots [2]. In addition, a biohybrid composition is aimed at biodegradability in medical applications. Coated magnetite suspension endowed not only magnetic responsivity but also magneto resonance (MR) contrasts. Because of the coating thickness, biodegradability was investigated by varying the morphology at 37 °C incubation based on the temporal condition of dipping. In addition, the navigation performance was demonstrated through an in vivo test in rodent stomachs. MR imaging was achieved because of the innate bacterial fluorescence properties. However, cessation of imaging in the deep organs remained a challenging issue. A Helmholtz coil system has limited penetration of a magnetic field induced by the low magnitude of the magnetic field despite the advantage of three axes of precise control in the magnetic field.

The rolling motility of the helical geometry was easily controlled through linear translational movements of the permanent magnets in dry environments [7]. Helical geometry is an effective means of reducing rolling resistance because of its lower mass, particularly when compared to the higher mass of cylindrical geometry. A 3D helical structure was fabricated using the following two-step process: a polymeric composite was formed by mixing soft magnetic iron particles (Fe) and polydimethylsiloxane (PDMS) and then molded to a pre-cured film (Figure 3b). PDMS is an optically clear and non-toxic thermoset elastomer whose liquid resin is commonly used for molding. Helix construction is crucial because the rolling motility of 3D helical soft robots can accomplish novel tasks such as uphill climbing even under a low magnetic flux density of 110 mT (Figure 3(bii)), whereas a two-dimensional (2D) composite film is only capable of being dragged using sliding resistance. Climbing was also demonstrated on discrete walls up to one-third the diameter of the body (Figure 3(biii)). The motion angle is parallel to the helix angle in the helical geometry, as reported by Wie et al., with light-induced rolling of helical azobenzene-functionalized liquid crystalline polymer networks (azo-LCNs) [14]. When magnetic momentum exceeds the rolling resistance and inertia, the rolling motility of magnetic soft robots emerges. Using permanent magnets, Park et al. achieved linear translational motility of helical magnetic composites by varying the magnetic velocity and magnetic flux density. 

The crawling motility of a multi-legged geometry solved a challenging issue regarding adaptability under wet and dry conditions, using 3-DoFs manipulator to control the position of permanent magnets. The leg geometry was prepared by the assembly phenomenon of magnetic polymeric composites along a magnetic flux (Figure 3c) [6]. Inspired by living organisms, the multi-legged geometry accomplished an ultrafast locomotion speed greater than 40 limb lengths/s. The legs reduced the friction force on the dry ground and effectively lifted the body. Soft robots move not only in an upward and downward direction but also in a left to right direction by a programmed external magnetic field. Individually different movements of each leg induce complicated movements. The flexible property of the body is advantageous to load objects up to 100 times its own weight (Figure 3(cii)). Furthermore, soft robots can cross a steep obstacle greater than 10 times their body height by standing erect at 90°.

### 2.2. Programming Alignments of Magnetic Components for Single-Modal Actuation

Programming spatial alignments of a magnetic component leads to selective locomotion in parts and the preparation of 3D robot geometry. Magnetic particles including magnetite (Fe_3_O_4_) or neodymium particle (NdFeB) are aligned like a chain in a liquid resin along the external magnetic field to minimize magnetic dipole interaction energy. If the aligned particles are solidified in polymer matrices, the chain-like particles move toward the external magnetic field and induce actuation of the entire structure. An ultraviolet (UV)-curable hydrogel or a photoresist polymer were utilized for matrices such as poly (N-isopropylacrylamide) (PNIPAM) and poly (ethylene glycol diacrylate) (PEGDA) or SU-8. PEGDA and PNIPAM are commonly used hydrogels favored for their low toxicity. Light-induced crosslinking is essential to delicately and selectively program magneto-alignments within polymer matrices. Prepolymer states of composites require a rapid process time and strong intensity of light for photo-polymerization to avoid magnetic particle agglomeration. 

**Figure 4:**
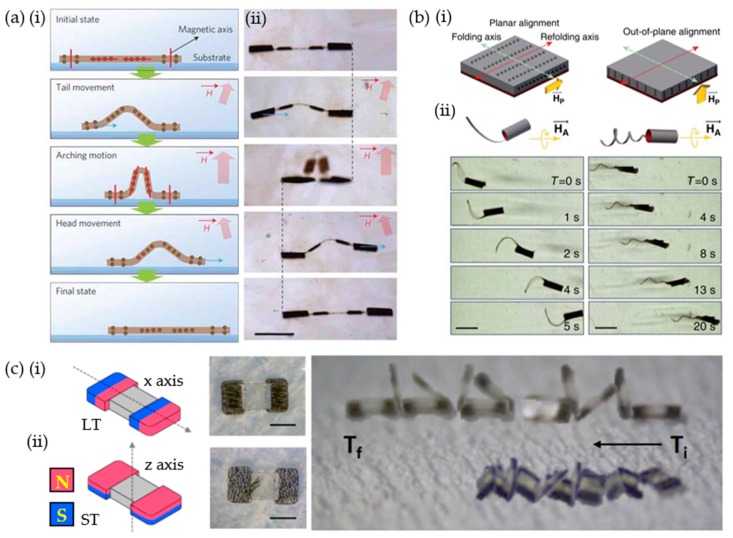
Directional motion induced by aligning magnetic components within a UV-curable polymer of soft robots. (**a**) (**i**) Schematic depiction of crawling motion, and (**ii**) optical snapshot images, including differently programmed head, tail (vertical alignments), and bodies (horizontal alignments) (scale bar: 100 μm). (**b**) (**i**) Preparation strategies for folding of a bilayer hydrogel film including two orthogonal magneto-alignments, and (**ii**) a time-lapse image of its swimming motility (scale bar: 2 mm). (**c**) (**i**) Tumbling locomotion of axial alignments of the x-axis, and (**ii**) z-axis for lengthwise tumbling (LT) and sideways tumbling (ST), respectively (scale bar: 300 μm). (**a**) Reproduced with permission [5]. Copyright 2011, Nature Publishing Group.

Crawling motility was achieved by sequential locomotion through partially different alignments of segments inducing an arching motion and forward movements (Figure 4(ai,aii) [5]. Superparamagnetic magnetite particles were aligned along the magnetic flux produced by permanent magnets as horizontal (bodies) and vertical (head and tail) alignments. Then, spatially resolved selective curing was implemented using a spatial light modulator (SLM) as the computational process. When the magnetic field was applied toward the upper right side, the tail of the soft robots was dragged forward. The magnetic field was changed to an upright direction and the two body sectors in the middle were deformed to an arch-like structure to arrange magnetic components parallel to the external magnetic field. Then, the upper left magnetic field induced forward head movement (i.e., a crawling motion). Swimming motility was also demonstrated through programming alignments of magnetic components by Nelson et al. Soft micromachines had orthogonal alignments of magnetite particles, namely, particles out-of-plane (supporting layer, upper part) and in-plane (responsive layer, lower part) (Figure 4(bi)) [4]. The programmed film was folded to form a tube-like geometry through the swelling properties of the hydrogel polymer, resulting in radial alignments of magnetic particles. Corkscrew-like rotating actuation was performed by radial alignment under a rotating magnetic field of 10 mT. In addition, aligned magnetite particles in the supporting layer also acted as reinforcing additives for a stiffness gradient. The folding axis of the tube was determined by the perpendicular direction of the aligned axis. Thermal conditions induced the folding and refolding phenomenon. This is because hydrogels swell below the low critical solution temperature (LCST) and shrink above the LCST (i.e., ~32 °C for PNIPAM) [65]. The swelling was also controlled for preferential folding toward a non-swelling PEGDA supporting layer against the swelling response of the PNIPAM layer. A helical tail was prepared by a folding mechanism perpendicular to the diagonal alignments and was compared to a planar tail to evaluate the swimming performance (Figure 4(bii)). The planar flagellum was shown to be propelled three times faster than the helical tail. This study illustrates the importance of stiffness gradients for the self-folding of 2D film. Furthermore, investigation of the curvy geometry suggests a strategy to develop the complicated geometry of soft robots.

Tumbling locomotion was devised by the morphology of segments of the polymeric composite body and magnetically inert polymer bridge. Polymeric composites of SU-8 and neodymium particles had positional alignments for magneto-motility [8]. The x and z axes were aligned and aimed for lengthwise tumbling (LT) (i) and sideways tumbling (ST) (ii) (Figure 4c). Hard magnetic particles had a merit of effective locomotion induced by a higher magnetization value at a lower magnetic flux density as compared to that of the soft magnetic particles. Initially, the middle bridge section was cured with a photomask, and then the composites were cast to both sides. The UV-curing process followed with a programming alignment of magnetic components using permanent magnets. Tumbling modes were demonstrated on rough substrates and inclined surfaces of 0°–60°. Although the geometry was constrained to a rectangle, the tumbling motility could achieve complex navigation including even a curvy route. The remaining problems were to minimize slip with substrates and hooking action on sharp corners during a curvy maneuver.

### 2.3. Programming Polarity of Magnetic Components for Multi-Modal Actuation

Alignment of magnetic dipole moments suggests a remarkable approach for multimodal locomotion under a low external magnetic field by programming the magnetization profile of hard magnetic materials based on high remanence characteristics. Magnetic moments of atoms in hard magnetic particles such as neodymium (NdFeB) microparticles are highly aligned under a few T of a magnetic field because the hard magnets feature high coercivity. However, magnetized hard magnetic particles yield magnetic polar directions after an external magnetic field is removed because of the high residual hysteresis. Tens of mT of a magnetic field are required to actuate magnetic soft robots compared to those composed of soft magnetic materials. The direction of the magnetic dipole moments is programmed when the magnetic particles are confined in cured polymer matrices, whereas the particle alignments are sequentially accompanied by rapid photopolymerization and masking techniques to prevent particle agglomeration in the prepolymer. Different polar directions within the soft robots cause partial deformation and actuation when the magnitude and directions of the applied magnetic field are varied. 

**Figure 5:**
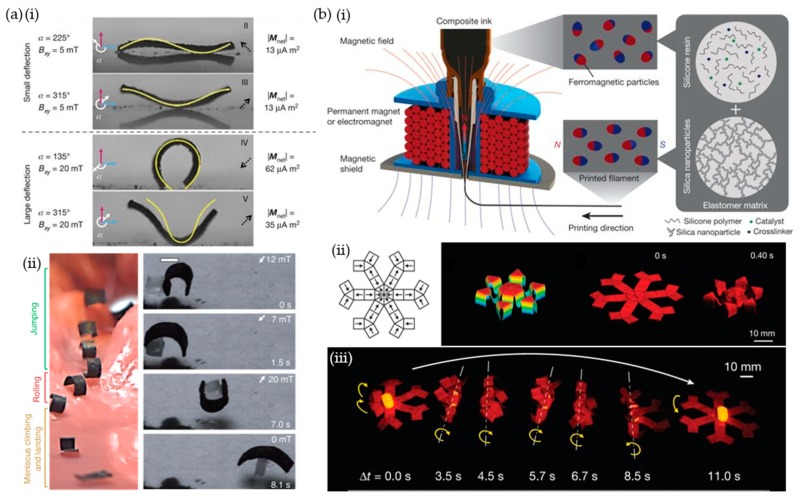
Multimodal magneto-actuation through multiple magneto-alignments in single soft robots. (**a**) (**i**) Reconfigurable soft body embedded with a counterclockwise alignment of polarity. (**ii**) Navigation across a synthetic stomach phantom and cargo transportation through multimodal locomotion (scale bars: 1 mm). (**b**) (**i**) 3D printing process using composite inks composed of uniaxial reoriented magnetic particles under a magnetic field. (**ii**) Printing design of magnetic polarity with a top-down view and actuation of the printed hexapedal and auxetic structure. The height of the auxetic 3D structure is 5 mm at the bottom of the panel. (**iii**) Carrying a pharmaceutical pill with a six-legged structure. (**a**) was used with permission [9]. Copyright 2018, Nature Publishing Group. (**b**) Reproduced with permission [10]. Copyright 2018, Nature Publishing Group.

Sitti et al. reported the following six locomotive modes from a single rectangular film as a strategy for controlling soft robots in diverse terrains: swimming, climbing, rolling, walking, jumping, and crawling [9]. A soft body was prepared using polymer composites of thermo-cured resin (Ecoflex10) and neodymium microparticles. Ecoflex has low density (1.04 g/cm^3^) and is easy to handle for molding techniques. When the composite film was wrapped around a cylindrical stick, particles were magnetized with a counterclockwise polarity under a horizontal magnetic field of 1.7 T. The curvature of the soft robot was sensitively manipulated based on the magnitude and direction of the magnetic field through the electromagnet. Deflection of sine, cosine, C, and V shapes could be achieved under a magnetic field of less than 20 mT (Figure 5(ai)). Meniscus climbing, landing, and swimming modes in water can be transited into rolling, walking, crawling, and jumping motions in land environments. The multimodal locomotion facilitates navigation across a surgical stomach phantom and cargo transportation (Figure 5(aii)).

Zhao et al. reported programmable multidirectional polarity for 2D and 3D structures by 3D printing composite inks [10]. The composite ink was composed of neodymium microparticles and silica nanoparticles dispersed in elastomer blends (SE1700 and Ecoflex30) and magnetized under 2.7 T. SE1700-grade silicone resin and silica nanoparticles were adopted for appropriate viscosity for 3D printing instead of Sylgard184-grade resin generally employed in the field of soft robots. The hard magnets were reoriented along the parallel direction of the flowing ink under a magnetic field of 50 mT at the tip using permanent magnets or electromagnets (Figure 5(bi)). Polarity morphing was readily imparted because the polar direction formed along the printing direction. The hexapedal 2D structure was folded under a perpendicular magnetic field at 200 mT. In addition, shrinkage with a negative Poisson’s ratio in the 3D auxetic structure was generated under the parallel direction of the magnetic field at 200 mT (Figure 5(bii)). Because of the aligned magnetic moments, the transportable functionality of holding and carrying objects was achieved through a simple rotation of a permanent magnet (Figure 5(biii)).

## 3. Liquid Crystalline Polymers

Liquid crystal materials present a unique phase behavior between a conventional solid and liquid because of their electrical and geometrical anisotropic characteristics such as a rod-like or discotic molecular structure, strong dipoles, and/or easily polarizable substituents. Liquid crystal polymer networks are synthesized by crosslinking reactive-mesogenic monomers and by maintaining the ordered structure of the anisotropic liquid crystal phase. In particular, crosslinked liquid crystal polymers have a wide range of moduli from approximately 0.1–5 MPa (low crosslink density liquid crystal elastomer; LCE) to 1–2 GPa (densely crosslinked liquid crystal network; LCN). In a nematic liquid crystalline polymer, the orientational order acts as an external body stress leading to elastic strain, whereas mechanical strain acts as an external field that aligns the mesogenic molecules [23]. Thus, a reduction in the degree of orientational order causes a mechanical strain of approximately 5% (LCN) to 90% (LCE) change upon heating. In general, photoisomerization of the dissolved dye [66] and thermal heating [67] scatter the molecules into a randomly oriented state in a liquid crystalline polymeric system. Upon heating, liquid crystal polymers undergo a nematic-paranematic [68] isotropic [69] phase transition, converting the material from an ordered to a disordered state. In light-responsive systems such as azobenzene-doped liquid crystal polymers, the order–disorder transition can be induced by breaking the molecular alignment via trans-cis photoisomerization of azobenzene moieties. 

**Figure 6:**
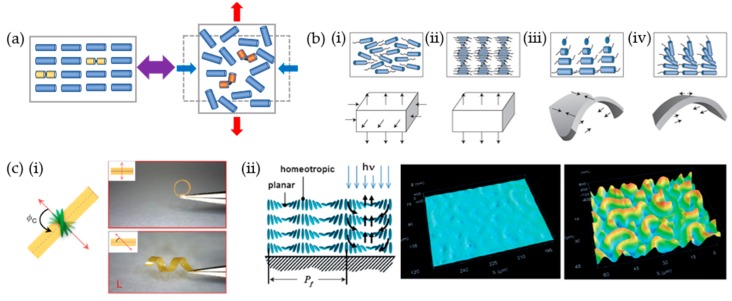
Principles of shape morphing for liquid crystalline polymers. (**a**) Schematic of the light-induced shape change of a liquid crystalline polymer resulting from the order–disorder transition. (**b**) Representation of the dimension changes in liquid crystalline polymers through order–disorder transition upon exposure to external stimuli: (**i**) uniaxial, (**ii**) cholesteric, (**iii**) twisted nematic, and (**iv**) hybrid geometry for liquid crystal polymers. (**c**) Molecular alignment-dependent actuations: (**i**) Bending and twisting depends on offsetting (*Φ_c_*) the nematic director of the molecule at midplane (red arrow) and the cutting direction (dotted line), (**ii**) 3D image of a dynamic fingerprint under UV exposure with a homeotropic to planar alignment. (**b**) Reproduced with permission [54]. Copyright 2015, Nature Publishing Group. (**c**) (**i**) Reproduced with permission [70]. Copyright 2014, Nature Publishing Group. (**ii**) Reproduced with permission [71]. Copyright 2014, Nature Publishing Group.

As a result of the decreased degree of order through external stimuli, the material undergoes shape morphing (contraction and expansion) in which the specific geometry of the deformation is determined by the distribution of the orientational direction within the material (Figure 6a). Thus, various physical approaches have been used to pattern the material structure and molecular alignment and obtain diverse and complex deformation/actuation modes. As shown in Figure 6b, the flat film bends into diverse shapes as a variety of molecular orientations across the film thickness [54]. Figure 6(ci) shows the actuation modes when the molecular alignments in the liquid crystalline polymers are varied [70]. As an example, the planar-to-homeotropic aligned molecules lead to complex forms of the film, which results in fingerprint-like shapes (Figure 6(cii)) [71]. This dynamic fingerprint-like surface can control the morphology under UV exposure and also modulate the friction. Furthermore, a complicated pattern of molecular distribution is needed for the 3D complex deformation. 3D printing [72,73,74] and lithography [75,76] are examples of technologies developed to create 3D polymer structures with sophisticated control over deformation. Moving beyond simple, light-driven motion of soft robots, the main difficulty is how to generate and regulate multiple shapes in a single robot.

### 3.1. Walking and Crawling Motion of Liquid Crystalline Polymeric Soft Robots

**Figure 7:**
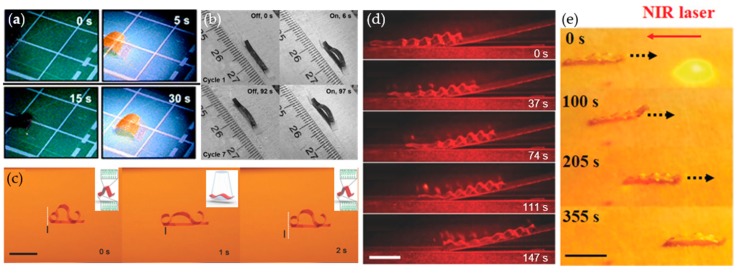
Crawling of liquid crystalline polymeric soft robots. (**a**) Photo-induced inchworm walking of the liquid crystalline polymer film by alternate irradiation with UV (366 nm, 240 mW∙cm^−2^) and visible light (>540 nm, 129 mW∙cm^−2^) at room temperature. (**b**) A light-driven caterpillar-inspired soft robot with alternated hybrid alignment (inset image). The initial bent shape deforms to flat geometry under visible light (448 nm, 150 mW∙cm^−2^). (**c**) Near-infrared (NIR)-light-driven walking carbon nanotube-liquid crystalline elastomer (CNT-LCE)/silicon bilayer soft robot. (**d**) Climbing a hill using the crawling motion of the soft robot. (**e**) Crawling of the wrinkle-shaped soft robot against the laser scanning direction (NIR, 980 nm, 4.5 W∙cm^−2^). The top of film was alternately crosslinked/decrosslinked, whereas the bottom of the film was fully decrosslinked. (**a**) Reproduced with permission [12]. Copyright 2009, Royal Society of Chemistry. (**b**) Reproduced with permission [13]. Copyright 2017, Wiley-VCH. (**c**) Reproduced with permission [20]. Copyright 2013, Wiley-VCH. (**d**) Reproduced with permission [21]. Copyright 2016, Wiley-VCH. (**e**) Reproduced with permission [22]. Copyright 2019, Wiley-VCH.

Actuation of a liquid crystalline polymeric soft robot is driven by directional contraction and expansion based on the orientation of the liquid crystalline molecules. A walking motion using the contraction and expansion between the legs is the simplest movement of a liquid crystal polymeric soft robot. Ikeda et al. demonstrated an inchworm walking motion [12] by employing alternative irradiation of UV and visible light, on a polyethylene-liquid crystalline elastomer (PE-LCE) bi-layered soft robot (Figure 7a). The PE-LCE laminated film underwent bending because of the mismatch of the coefficient of thermal expansion between the two layers. Azobenzene and the liquid crystal mesogens prepared in the smectic phase showed a higher-order along the long axis of the film. Upon UV light irradiation, soft robots expand to a flat shape and recover their initial bent shape under visible light irradiation. When the preceding principles were applied, unidirectional inchworm walking motion of the soft robot was performed by adapting the following asymmetric end shapes: flat and sharp edges on the front and back, respectively. Afterward, a monolithic liquid crystal soft robot walker, based on alternately hybrid-aligned (planar to homeotropic aligned in the thickness direction), was demonstrated by Priimagi et al. [13]. The hybrid-aligned azobenzene-doped liquid crystal bent under light irradiation because of its alignment distribution in the thickness direction. Figure 7b shows three alternated hybrid-aligned segments and a Ω-shaped caterpillar robot walk toward temporally modulated visible light (488 nm, 150 mW∙cm^−2^). In addition, the alternating molecular alignment of the monolithic liquid crystalline polymer enabled soft robot crawling motion. Furthermore, Kohlmeyer et al. attempted to implement the walking motion through infrared (IR) light using carbon nanotubes (CNTs) as IR activation fillers, with LCE as the matrix/silicon bilayer (Figure 7c) [20]. Under NIR irradiation, the CNTs absorbed the light and conducted the heat to the LCE sequentially, which induced contraction by the loss of the orientational order.

Crawling motions of soft robots are shown in Figure 7d,e. Wiersma and Wasylczyk demonstrated the movement of a larva-like soft robot during various tasks such as climbing a hill (Figure 7d) [21], squeezing through a slit, and moving a metal cylinder. These were accomplished by patterning the molecular orientation and through laser beam scanning. In addition, Zhao et al. employed a selective crosslinking–decrosslinking method using the anthracene moiety in LCNs (Figure 7e) [22]. Anthracene is known for reversible photodimerization (365 nm) and photocleavage (254 nm) of dimers under UV irradiation. Unidirectionally aligned anthracene functionalized LCN films can be partially decrosslinked through irradiation of a patterned 254 nm UV light. These decrosslinked sites can act as flexible hinges. A liquid crystalline soft robot with an alternated crosslinked–decrosslinked top surface and a fully decrosslinked bottom layer performs a crawling motion by utilizing the heat from an NIR light (Figure 7e).

### 3.2. Biomimetic Swimming Motion of Liquid Crystalline Polymeric Soft Robots

**Figure 8:**
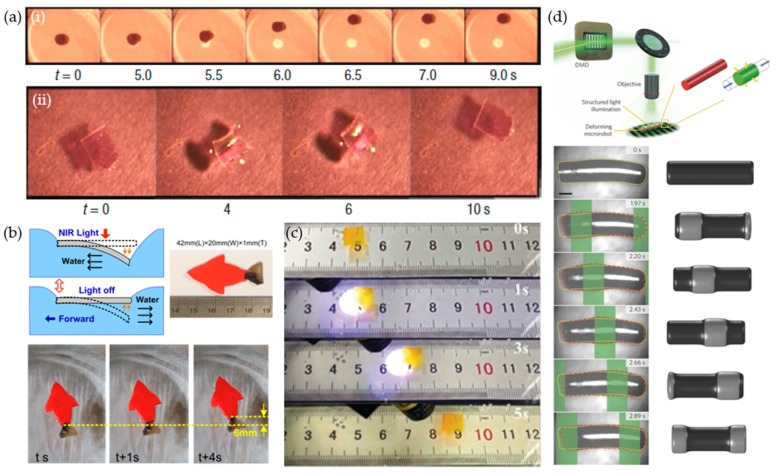
Swimming motion of liquid crystalline polymeric soft robots. (**a**) Photo-induced actuation of liquid crystal elastomer (LCE) on the liquid surface: (**i**) disk-shaped (5 mm in diameter, 0.32 mm in thickness) LCE on water swimming away from light (Ar^+^ ion laser, 495 nm, 1.1 W cm^−2^), (**ii**) rectangular-shaped LCE in ethylene glycol that folds and then swims away from the light. (**b**) Soft robotic swimmer based on polydopamine-coated LCE. Schematics of the bending/unbending of the soft robotic swimmer (up) and swimming motion of the soft robot near the air–water interface (down). (**c**) Swimming motion of an azobenzene-containing liquid crystal network (LCN)-Kapton bilayer swimmer. (**d**) A dynamic light field from a digital micromirror device is projected onto the liquid crystal polymeric soft robot (left), which propels itself in liquid through traveling-wave deformation (right). (**a**) Reproduced with permission [23]. Copyright 2004, Nature Publishing Group. (**b**) Reproduced with permission [24]. Copyright 2018, American Chemical Society. (**c**) Reproduced with permission [25]. Copyright 2019, Wiley-VCH. (**d**) Reproduced with permission [26]. Copyright 2016, Nature Publishing Group.

In addition to walking motions, biomimetic swimming motions at liquid–air interfaces or underwater represent an advanced field in soft robots. The swimming motion of a liquid crystal polymer was first documented by Palffy-Muhoray et al. [23]. A disk-shaped azobenzene-doped LCE swimmer was floated on ethylene glycol (Figure 8(ai)) and water (Figure 8(aii)). Photo-induced locomotion was performed using an Ar^+^ ion laser (514 nm, 1.1 W cm^−1^). This locomotion was essentially the same in the presence of a surfactant soap. Because of the shape-morphing dependence between the molecular alignment and strain response, a polydopamine-coated LCE swimmer was reported for swimming direction control and rapid movement (Figure 8b) [24]. Polydopamine was coated on one side of the LCE film, and the LCE film was heated through the photothermal effect. Because the polydopamine has an excellent photothermal effect and strong adhesive property, a polydopamine-coated LCE swimmer is well driven on air–water surfaces. As shown in Figure 8c, Yu et al. reported an LCN-polyimide bilayer swimming robot [25], which exhibited rapid swimming motion similar to a dolphin with on-demand directional control. This was possible because the rigid LCN-based material can perform fast recovery as compared to that of soft LCE material. The underwater swimming motion of a soft robot is important because of its possible use in biological and medical applications. Fischer et al. demonstrated the self-propelled swimming capabilities of an LCE micro-robot in 2016 (Figure 8d) [26]. This swimming microbot had many degrees of freedom in motion because the intrabody shape change of the soft robot was controlled by structured and programmed light irradiation, providing control in terms of the pathway, speed, and location.

### 3.3. Rolling Motion of Liquid Crystalline Polymeric Soft Robots

**Figure 9:**
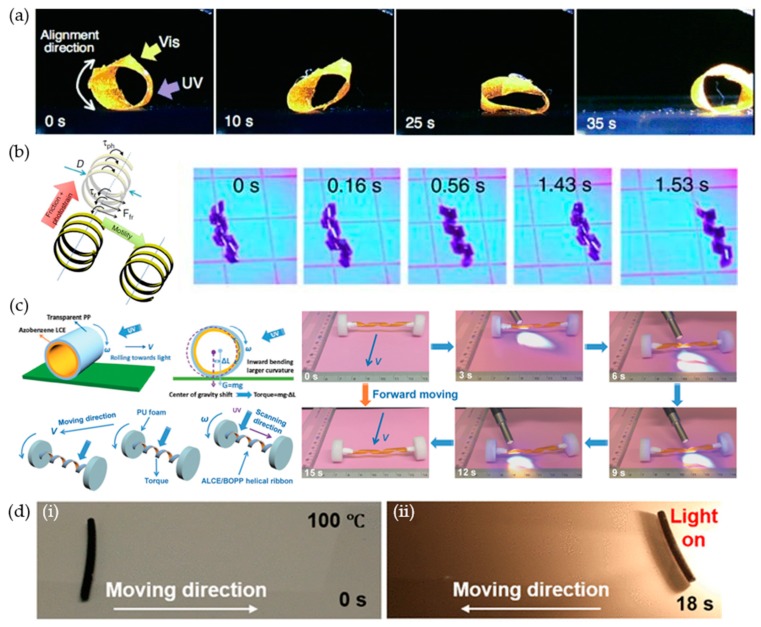
Rolling motions of liquid crystalline polymeric soft robots. (**a**) Photo-induced rolling motion of a ring-shaped LCE film. The LCE ring was irradiated through UV (366 nm, 200 mW∙cm^−2^) and visible light (>500 nm, 120 mW∙cm^−2^). (**b**) Rapid translation of a helical liquid crystalline polymer under continuous light illumination. (**c**) Light-driven forward movement of a wheel supported LCE-Polypropylene (PP) vehicle. The LCE-PP helical ribbon was configured with a spring-like motor. (**d**) Rolling motion of an LCE-carbon nanotube (CNT) rod: (**i**) rolling of the LCE-CNT rod on a hot surface, (**ii**) light-induced reverse rolling of an LCE-CNT composite rod on a flat hot surface. (**a**) Reproduced with permission [27]. Copyright 2008, Wiley-VCH. (**c**) Reproduced with permission [15]. Copyright 2017, Wiley-VCH. (**d**) Reproduced with permission [16]. Copyright 2018, American Chemical Society.

Rolling is an effective in-plane locomotive mode because the rolling resistance is significantly lower than the sliding friction. The first rolling motion of a liquid crystalline polymer was demonstrated by Yamada et al. [27]. As shown in Figure 9a, an LCE ring was prepared by connecting both ends of the film. Azobenzene mesogens were aligned in a circumferential direction. Upon exposure to UV light on the underside and visible light on the upside of the ring, the LCE rolled in the irradiation direction. Rolling of a helical azo-LCN under continuous irradiation of a temporally and spatially stationary broadband UV-vis light, was demonstrated by Wie et al. (Figure 9b) [14]. When the sample was exposed to light, the upper portion of the azo-LCN film that was in the optical path attempted to adopt a greater twist in contrast to the portion in which the light was shadowed. The asymmetric photo-strain between the top and bottom of the helical coil induced torque to overcome the rolling resistance. In addition, broadband light also regenerated the active azobenzene molecules through cis-trans back isomerization, and the rolling persisted as long as the light irradiation continued. Another example of light-induced rolling motion was documented by Lu et al. [15]. The azobenzene liquid crystalline polymer was laminated with polypropylene and subsequently fabricated into a spring shape by twisting the film around a cylindrical rod. The motion behavior of the light-driven rolling was determined by the location of the LCE film (inside or outside): rolling away or toward the light when the LCE was outside or inside, respectively. When the ends of the helical ribbon were linked with two wheels, the rolling resistance was reduced. This enabled the soft robot to convert light into a rolling motion, more effectively (Figure 9c) [15]. Photothermal heating also generated a rolling motion in liquid crystalline materials. Ahn et al. reported the rolling motion of a CNT-LCE composite rod (Figure 9d) [16]. When the CNT-LCE rod was on a heated surface, asymmetric heat distribution, high temperature on the bottom, and low temperature on the top induced a volumetric mismatch and generated a gravitational force that caused the LCE rod to roll. Subsequently, light exposure on the CNT-LCE composite generated heat at the top portion of the rod and induced the rod’s rolling motion.

### 3.4. Wave and Jumping Motions of Liquid Crystalline Polymeric Soft Robots

**Figure 10:**
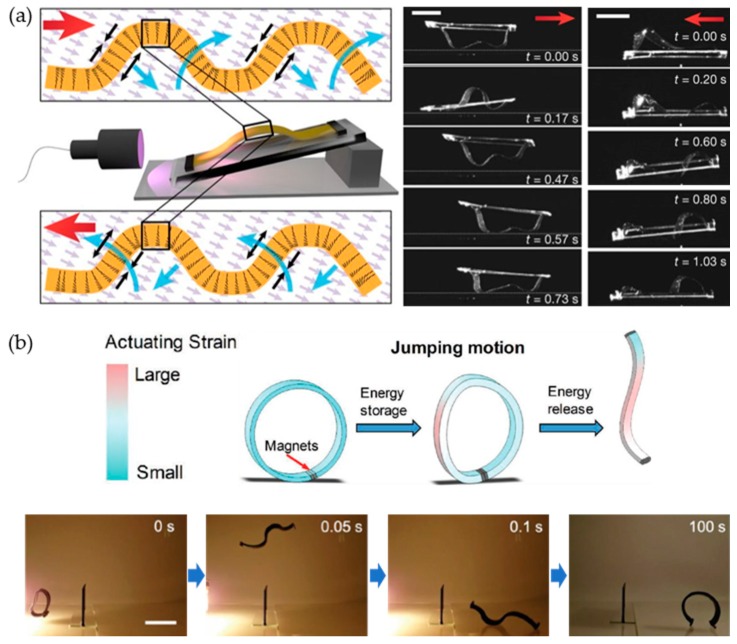
(**a**) Making waves in LCN films. (**b**) Jumping of CNT-LCE composites with magnets on both ends. (**a**) Reproduced with permission [17]. Copyright 2017, Nature Publishing Group. (**b**) Reproduced with permission [18]. Copyright 2019, Wiley-VCH.

Other examples of unique motions of liquid crystalline materials were achieved by a confined structure. Gelebart et al. demonstrated the waving motion of a liquid crystal elastomeric film by confining both ends (Figure 10a) [17]. Liquid crystalline molecules and light-responsive azo molecules were aligned in a hybrid geometry—homeotropic on the surface and planar on the other. A buckled wave shape was created with the limited movements of fixed ends of the film. When irradiated by light from the side, a waving motion was initiated and repeatedly propagated. Because the azo-derivative has a very short half-life, the film can quickly recover from shadowed areas and maintain waving until the light turns off (Figure 10(ai)). Finally, LCN was attached to a non-responsive plastic frame and the photomobile device moved while the wave was traveling through the sample. The waving direction was controlled by the film orientation. Recently, a jumping motion was achieved by Ahn et al. by attaching both ends of the CNT-LCE composite film using a magnet (Figure 10b) [18]. The CNTs generated the photothermal heat on the LCE matrix under light exposure (halogen lamp, 1.57 W/cm^2^) and contracted in the alignment direction. Because the magnet holds both ends of the film, energy from the photothermally generated strain was stored in the system. When light irradiation was sustained and the stored elastic energy exceeded the force of the magnet, the rapidly releasing energy was converted to a jumping motion.

## 4. Hydrogel-Based Soft Robots

**Figure 11:**
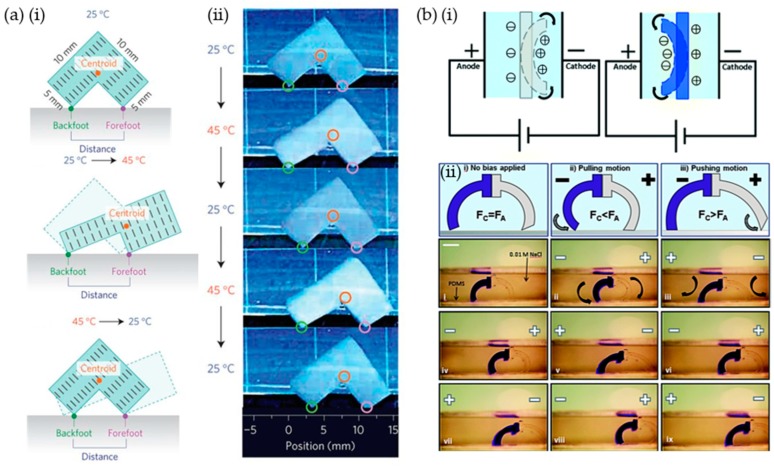
Actuation of hydrogel-based soft robots. (**a**) (**i**) Schematic of the ideal processing mechanism of anisotropic thermal expansion of a hydrogel-based soft robot (5 mm thick) from electrostatic repulsion of titanium nanosheet (TiNS) in dehydrated hydrogel, (**ii**) thermo-responsive actuation of an electrostatically anisotropic hydrogel soft robot. Alternate heating and cooling was conducted between 25 °C and 45 °C at a rate of 0.1 °C/s. (**b**) (**i**) Scheme of the bending direction of electro-active materials; acrylamide (AAm)/sodium acrylated (NaAc) copolymer (light gray) and AAm/quaternized dimethylaminoethyl methacrylate (DMAEMA-Q) copolymer (blue), (**ii**) electro-actuated hydrogel walker in 0.01 M NaCl composed of 50% NaAc and 30% DMAEMA-Q legs and an applied field of 5 V/cm (scale bar: 5 mm). (**a**) Reproduced with permission [28]. Copyright 2015, Nature Publishing Group. (**b**) Reproduced with permission [40]. Copyright 2014, Royal Society of Chemical.

Hydrogel-based soft robots are commonly manipulated by heat [29,30,31,32,33] and electric fields [39,40,41,42]. As previously mentioned, the volume and shape of a hydrogel can be changed when heated above the LCST, making the hydrogel a useful component in soft robots. For example, Aida et al. developed a soft robot based on an anisotropic thermal-responsive PNIPAM gel using a titanium nanosheet (TiNS) as a filler [28]. Gel liberated water molecules from the network and recovered their intrinsic electrostatic permittivity, thereby enhancing the electrostatic repulsion between the cofacially oriented TiNS’s through heating above the LCST (~32 °C). Enhanced electrostatic repulsion induced anisotropic thermal expansion in the orthogonal direction to the TiNS plane. As shown in Figure 11a, an L-shaped symmetric hydrogel soft robot has unidirectional procession through oscillatory change in the temperature.

In addition, hydrogel, which is composed of ionic materials, can be manipulated by applying electrical fields [39,40,41,42]. Electrical fields asymmetrically distribute mobile ions that create osmotic pressure differences between the ionic hydrogel and solution, which swell and deform the hydrogels. For example, Velev et al. demonstrated electro-actuated hydrogel walkers with dual responsive legs [40]. As shown in Figure 11b, they prepared two legs composed of an acrylamide (AAm)/sodium acrylated (NaAc) copolymer (shown in light gray) and AAm/quaternized dimethylaminoethyl methacrylate (DMAEMA-Q) copolymer (shown in blue), which were cationic (light gray) and anionic (blue) hydrogels. The hydrogel walker is composed of two legs which had different swelling directions that were opposite to each pole in an aqueous solution. For unidirectional continuous motion, each leg had a different tip in order to obtain an asymmetric friction coefficient, and an electric field was alternatively applied. Initially, the pole of the applied electric field coincided with the soft robot pole. This coincidence caused the legs to bend inside the soft robot. The anionic leg (light gray) had a larger contact area, with a PDMS substrate, as compared to the cationic leg (blue). This asymmetrical contact area caused the walker to move to the right. In pushing motion step, switching the field caused the anionic leg to stretch toward the cathode due to the higher friction force of the cationic leg, which anchored anionic leg on the substrate. Therefore, the cationic leg effectively pushed the gel walker forward. This repetitive cycle enabled the unidirectional perpetual motion of the hydrogel walker.

## 5. Chemical-Based Soft Robots: Solvent and Chemical Reaction

**Figure 12:**
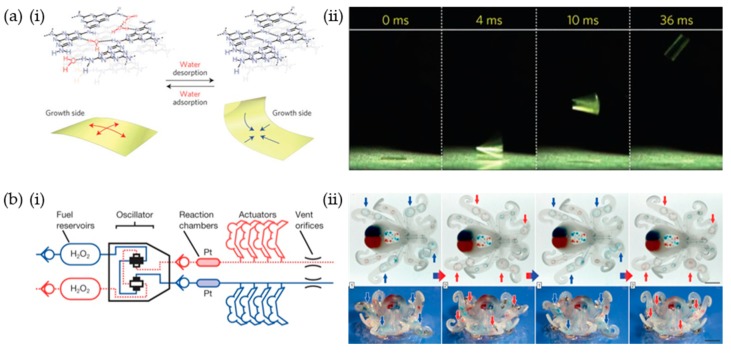
(**a**) (**i**) Schematic of moisture adsorption- and desorption-driven actuation of π-stacked carbon nitride polymer (CNP) film, (**ii**) scheme and high-speed snapshot of the jumping motion of a CNP film under UV (365 nm) irradiation. (**b**) (**i**) Scheme of the octobot system. Oxygen propellant from the decomposition of the H_2_O_2_ fuel induced pneumatic actuation of the octobot, (**ii**) monopropellant decomposition-powered actuation of the octobot (scale bar: 10 mm). (**a**) Reproduced with permission [37]. Copyright 2016, Nature Publishing Group. (**b**) Reproduced with permission [43]. Copyright 2016, Nature Publishing Group.

The actuation of solvent-responsive soft robots is driven by the swelling of polymers by solvent adsorption and deswelling of polymers by desorption under dry conditions [34,35,36,37,38]. For example, Aida et al. prepared a jumping soft robot based on a π-stacked carbon nitride polymer (CNP) film [37]. As shown in Figure 12a, CNP film effectively adsorbs water molecules by hydrogen bonding to unreacted nitrogen. In this state, the film has a straight form. After the water in the CNP film was removed by heating or using dry air, the film bent because of the asymmetric structure. The large and rapid actuation of the CNP film enabled jumping through the photothermal effect from irradiation with UV. In addition, Kim et al. prepared a hygroscopic responsive soft robot they called a hygrobot [38]. The hygrobot was composed of an active/inactive layer with two legs. The dry bilayer was initially bent in a convex form downward. In this configuration, the end tip of the foreleg and the knee of the back leg were in contact with the substrate. The bilayer bent upward with swelling of the active layer, and the back-leg slid to the foreleg because the static friction coefficient of the end tip was higher than that of the knee when the environmental humidity increased. When moved back to the dry condition, the foreleg slid forward given the back leg was fixed. When the humidity cycle is repeated with a temporal humidity variation, directional locomotion of the hygrobot was achieved.

Pneumatic soft robots have successfully demonstrated various actuation modes such as crawlers [77,78], swimmers [79], and robust jumpers [80,81]. However, a rigid power supply and controller are tethered to the soft robots or on-board systems are used to control the aforementioned soft robots. To create fully soft and autonomous robots, Wood et al. developed an octopus-shaped autonomous pneumatic soft robot by implementing chemical decomposition of monopropellant fuels (Figure 12b) [43]. A microfluidic soft controller was embedded in the octopus-shaped soft robot, called an octobot, to supply pressure to the legs. The flow of fuel (hydrogen peroxide (H_2_O_2_)) was controlled by an oscillator that included a check valve to reach the reaction chamber. During this stage, the gas pressurized the microfluidic channel and deformed the legs from chemical deposition of the liquid fuel.

## 6. Outlook and Conclusions

In this review, we highlighted the recent examples of polymeric materials for untethered soft robots such as magnetic composites, light-responsive liquid crystalline materials, and various polymeric materials responsive to heat, solvents, and gases formed by chemical reactions. Two major themes were identified in the soft robotics research: diversification of soft robots in terms of motions and stimuli. Assorted movements such as rolling, jumping, and swimming were achieved by programming magnet particles within composites and molecular orientation of liquid crystalline materials. Examples of variegated stimuli included the study of temperature-induced walking motion with hydrogels containing titanium nanosheets, which generate anisotropic thermal expansion and electro-activity of dual responsive anionic and cationic hydrogels that respond to positive or negative charge. Other examples included CNP film jumping, hygrobot walking using moisture adsorption–desorption, and an octobot that uses pneumatic pressure generated by the decomposition of H_2_O_2_. Each method has advantages and disadvantages. For example, in the case of photoactive robots, the light propagation pathway to the robot should be secured. Magnetomotility was shown to have a distance limitation in terms of the controllable range because the magnetic field is considerably dependent on the distance from the source. In addition, creating a uniform magnetic field over a large area was found to be difficult. For solvent or water-driven systems, wet operating conditions hinder precise actuation control such as spatiotemporally-localized actuation or controlled magnitude of actuation. Ultimately, multi-responsive soft robotic systems are necessary to circumvent the limitations of stimuli and materials.

The ideal goal of a soft robot is to perform a variety of sophisticated movements with a large magnitude of both stress and strain responses, under simple external stimuli. In general, however, sophisticated and programmed external stimuli are required to manipulate soft robots consisting of isotropic materials. Conversely, programmable anisotropic materials enable soft robots to be easily regulated through simple stimulation. Even for programmable anisotropic materials, simple stimulation often results in the repetition of single-mode motility. Therefore, the combination of programmable materials and stimuli is essential to achieve wireless controlled soft robots capable of multi-modal locomotion, as exemplified by the recent report from Sitti et al. [9]. In addition to multi-modal motility under external stimuli, generating large stress is necessary to perform various functions. Simultaneous generation of large stress and strain responses is often limited by stress-strain correlations [82,83]. A good example of achieving both large strain and stress response is liquid crystal elastomers, which can lift 2700 times their weight through cooperative motions, as reported by White et al. [84]. For further research trends, soft robots will be able to achieve various motions by discrete stimuli in terms of multimodal actuations. The anisotropic factors of geometrical shape, material alignment, and material polarity play pivotal roles in regulating multifunctional soft robots. In addition, multi-stimuli response is needed to push the limits of one system to other stimuli. For example, if a soft robot can independently react to all orthogonal stimuli, one robot can have complex motions with multi-stimuli input, such as holding an object by light while spatially moving under a magnetic field. Furthermore, a motion tracking system will help complete complex movements of soft robot by providing instant feedback on the localized stimuli based on real-time positional information. Finally, we expect that the integrated motion-tracking and stimuli localization will be further refined through machine learning.

## Figures and Tables

**Table 1:** A brief summary of magnetic soft robots including motility, external environments, magnetic source for actuation, geometry, materials, fabrication method, advantages, and limitations.

Categories	Motility	Magnetic Source for Actuation	Geometry	Materials	Fabrication Method for Geometry	Advantages	Limitations
Programming geometry of robots for desired actuation	Swimming	Rotating magnetic field of Helmholtz coil	Helical coil	SU-8 or IP-L and Ni/Ti	DLW (dynamic light writing) [1]	High resolution morphology and transportability of cargo using carrier	Fragile body of matrix materials
				S. Platensis and magnetite	Dip coating [2]	Biocompatibility, biodegradability, and MR imaging functionality	
	Rolling	Linearly applied magnetic field of permanent magnets		PDMS (polydimethylsiloxane) and iron microparticles	Wrapping precured film around sticks [7]	Facile fabrication method, linear motility despite of helix geometry and climbing uphill and discrete walls	Difficult control of precise locomotion directions
	Crawling		Multi-legs with body		Assembly of uncured composite resin [6]	Facile fabrication method, fast locomotion speed, loading cargo and climbing uphill	
Programming alignments of magnetic components for single-modal actuation	Crawling	Linearly applied magnetic field of Helmholtz coil	Head and tail	PEGDA (poly ethylene glycol diacrylate) and silicon coated magnetite	SLM (spatial light modulator) with aligning of magnetic particles by permanent magnets [5]	Uncomplicated control of locomotion directions (forward/backward)	Multiple manipulation steps of magnetic field for one motion
	Swimming	Rotating magnetic field of Helmholtz coil		PEGDA (poly ethylene glycol diacrylate), PNIPAM (poly N-isopropylacrylamide) and magnetite	Lithography with alignment of magnetic particles by solenoid coil and folding/defolding of body by temperature variation [4]	Multiple stimuli responses and reversible shape morphing	Complicated fabrication steps
	Tumbling		Bridged body	SU-8 and NdFeB (neodymium) microparticles	Lithography with alignments of magnetic particles by permanent magnets [8]	Climbing uphill and steerability to desired positions	Fragile body of matrix materials
Programming polarity of magnetic components for multi-modal actuation	Multimodality (swimming, climbing, rolling, walking, jumping, crawling)	Linearly applied magnetic field of electromagnets and Helmholtz coil	Film	Ecoflex and NdFeB (neodymium) microparticles	Wrapping precured film around sticks and aligning of particle polarity by electromagnets [9]	Multimodal locomotion of a single body, transportability of cargo, and steerability to desired positions with precise regulation	Difficulty of remaining desired geometry without magnetic field
	Rolling/crawling /jumping	Linearly applied magnetic field of Helmholtz coil	Diverse structures (e.g., hexapedal body)	Ecoflex, PDMS and NdFeB (neodymium) microparticles	Inkjet printing and alignment of particle polarity by electromagnets [10]	Facile alignment process along printing directions, construction of diverse hierarchical three-dimensional structures	
